# Idiopathic severe aplastic anemia with a delayed response to immunosuppressive therapy: a case report

**DOI:** 10.1002/ccr3.1517

**Published:** 2018-04-10

**Authors:** Nujood Alzahrani, Nshwa Ashor, Traji Fathi, Dania Bukhari, Galila Zaher

**Affiliations:** ^1^ King Abdulaziz University Jeddah Saudi Arabia; ^2^ King Abdulaziz University Hospital Jeddah Saudi Arabia

**Keywords:** Antithymocyte globulin, aplastic anemia, immunosuppressive

## Abstract

Bone marrow transplantation is the definitive treatment of severe aplastic anemia; however, with the absence of this option, combined immunosuppressive therapy with antithymocyte globulin (ATG) and cyclosporine A is used as a first‐line therapy. This case report highlights the possible delay in response to ATG protocol in treating aplastic anemia.

## Introduction

Aplastic anemia (AA) is a bone marrow disorder characterized by peripheral pancytopenia and hypocellularity of the bone marrow 1. In the majority of cases, AA is due to autoimmune mechanisms that target the progenitor stem cells leading to pancytopenia. Furthermore, environmental exposures such as chemicals, drugs, viral infections, and endogenous antigens generated by genetically altered bone marrow cells can trigger an abnormal immune response 2. Aplastic anemia is classified into “non‐severe,” “severe,” and “very severe.” This classification is based on the degree of peripheral blood cytopenia, absolute reticulocyte count, and degree of bone marrow cellularity, as assessed using trephine biopsy 3.

Hematopoietic stem cell transplantation should be offered as the first line of treatment for patients with severe aplastic anemia who are <40 years of age and who have a HLA‐matched sibling donor 2. However, patients who lack a compatible donor are treated with immunosuppressive therapy (IST). In such cases, the treatment option includes antithymocyte globulin (ATG), cyclosporine A (CSA), and steroids 4. In this report, we present a case of a young woman diagnosed with idiopathic severe AA and discuss how she was successfully treated with IST containing rabbit ATG (rATG).

## Case History/Examination/Investigations

A 25‐year‐old female woman presented with a two‐week history of bruises on her lower limbs prior to presentation. The patient had no history of a previous similar occurrence, and she also denied epistaxis, gum bleeding, and a heavy menstrual bleeding. Associated symptoms such as fever and weight loss were absent. She denied using medications, receiving blood transfusions, or having a family member with bleeding disorders. She mentioned that she had two siblings.

On physical examination, the patient had stable vital signs, with unremarkable findings except for ecchymosis on her lower limbs. She was pale, but had no palpable lymphadenopathy, or hepatosplenomegaly. Her neurological examination results were normal. The initial complete blood count (CBC) revealed pancytopenia (Table 1). Renal and liver function test results, biochemical workup findings, and coagulation profile were all normal. Her peripheral blood smear showed no abnormal cells, no red cells fragmentation or polychromasia, and normal platelet size; however, the blood smear confirmed that the patient had pancytopenia.

**Table 1:** Complete blood count (CBC) results

Test name	Initial CBC	18 months later	Unit	Reference range
White blood cells (WBC)	1.90	4.25	K/*μ*L	4.5–11.5
Red blood cells (RBC)	3.75	2.47	M/*μ*L	4–5.40
Hemoglobin (Hb)	11	8.8	g/dL	12–15
Hematocrit (Hct)	30.6	26.9	%	35–49
Mean cell volume (MCV)	81.6	108.9	fL	80–94
Mean cell hemoglobin (MCH)	29.3	35.6	pg	32–36
Platelets	12	51	K/*μ*L	150–450
Reticulocyte absolute	0.020	0.128	%	0.5–2

Based on the findings mentioned above, aplastic anemia was suspected. The exclusion of other diseases was derived from the laboratory test findings. For example, normal vitamin B12 and folate levels ruled out megaloblastic anemia. The negative viral serology and autoimmune profile (Table 2) excluded viral infections and autoimmune diseases, respectively. A direct Coombs test yielded negative results ruling out Evan's syndrome and the absence of schizocytes in the blood smear along with the absence of the neurological defects and renal impairment ruled out thrombotic thrombocytopenic purpura (TTP).

**Table 2:** Autoimmune and viral serology profiles

Test name	Result	Reference range
ANA	Negative	
Anticardiolipin M	70	0–7 U/mL
Anticardiolipin G	<0.5	0–10 U/mL
AMA	Negative	
C‐ANCA	Negative	
P‐ANCA	Negative	
SMA	Mildly +	
CMV IgG	Positive	
EBV IgM	Negative	
HCV Antibodies	Negative	
HAV IgM	Negative	
HBsAg	Negative	
HIV	Negative	
VDRL	Negative	

ANA, antinuclear antibody; AMA, antimitochondrial antibody; SMA, smooth muscle antibody; CMV IgG, cytomegalovirus immunoglobulin G; EBV IgM, Epstein Barr virus immunoglobulin M; HCV, Hepatitis C; HAV, Hepatitis A; HBsAg, Hepatitis surface antigen; HIV, Human immunodeficiency virus; VDRL, Venereal Disease Research Laboratory.

Bone marrow aspiration was aparticulate and acellular; however, no abnormal cells were seen. Trephine bone marrow biopsy showed markedly hypocellular bone marrow with cellularity <5% and thickened trabeculae. Additionally, the marrow was replaced mainly with nonhematopoietic tissue, such as fat; the marrow was nearly absent of hematopoietic elements, which is consistent with severe aplastic anemia. Therefore, the diagnosis of idiopathic thrombocytopenic purpura was excluded, and we confirmed the diagnosis of idiopathic acquired severe aplastic anemia.

Immunophenotyping studies showed no evidence of leukemia, myelodysplasia, or paroxysmal nocturnal hemoglobinuria. To rule out any underlying malignancy, chest radiography and pan‐CT imaging were performed. Imaging findings were unremarkable except for a small right breast mass measuring 0.8 × 0.6 cm. Further, ultrasound imaging of the mass showed a morphologically benign solid mass of the breast with a BI‐RADS score of 3; fibroadenoma was the most likely diagnosis. Ultimately, the patient was diagnosed with idiopathic acquired severe aplastic anemia.

## Treatment

During her hospital stay, the patient received multiple platelets and packed red blood cells (PRBCs) transfusions to achieve a hemoglobin level of 80–100 g/L and platelet count of >10 × 10^9^ cells/L and up to 20 × 10^9^ cells/L when she was febrile according to the AABB guidelines 5. During this phase, HLA typing was performed for the patient and for her brothers and sisters to find a suitable donor for bone marrow transplantation. Although she had a matched HLA sibling donor for a bone marrow transplant, unfortunately, this option was not available in our hospital and the patient was not able to afford the procedure. After counseling, she was started on immunosuppressive therapy (IST). Family members were avoided as potential donors in any blood product transfusion. Leuko‐depleted blood products and single donor platelet transfusions were used whenever a blood transfusion was required. Following that, rabbit ATG was administered after a skin test for hypersensitivity. ATG is usually administered at a dose of 1.5 mg/kg over 4 h, daily for 4 days. As prophylaxis for serum sickness, prednisone 1 mg/kg was started on day 1 and continued for 2 weeks, and CSA was initiated on day 1 to a target trough level between 200 and 400 ng/mL, starting at a dose of 10 mg/kg per day. The platelet count was maintained at more than 20,000/*μ*L during the ATG administration period.

## Outcome and Follow‐up

The patient was discharged from the hospital on CSA and prednisolone. During follow‐up in the clinic, her CBC, renal, and liver function test results were monitored regularly, and her CSA level was monitored with a target level of 200–400 ng/mL 6. She required regular PRBCs and platelet transfusions, particularly during her menstrual period. To minimize heavy menstrual flow, she was started on the antifibrinolytic tranexamic acid 500 mg orally three times per day.

Within the following months, the patient had frequent visits to the emergency department (ED) presenting with symptoms related to anemia such as palpitations and shortness of breath or symptoms related to thrombocytopenia such as menorrhagia for which she received blood transfusions. Thus, she needed PRBC transfusions every 2 months. She started to respond to the treatment approximately 18 months after her first dose of CSA. Subsequently, she received fewer transfusions and her pancytopenia improved. The last CBC report is shown in Table 1.

## Discussion

Aplastic anemia is a blood disorder resulting from bone marrow failure. It is best characterized by peripheral pancytopenia and hypocellularity of the bone marrow 1. To diagnose severe aplastic anemia (SAA), two of the following criteria have to be met: a platelet count of <20 × 10^3^/mm^3^, an absolute neutrophil count (ANC) of <500/mm^3^, a reticulocyte count of <60 × 10^3^/mm^3^, and/or the presence of bone marrow cellularity of <30% 3.

Until the 1970s, severe aplastic anemia (SAA) was almost uniformly fatal, but currently, most patients can be effectively treated and can expect long‐term survival. In spite of this, making a diagnosis and selecting treatment options are not straightforward 6. Bone marrow transplantation is the definitive therapy; however, with the absence of this option, combined immunosuppressive therapy with ATG and cyclosporine A is used as a first‐line therapy 7. With regard to ATG, it can exert various effects on the immune system, including T‐cell depletion in the blood and peripheral tissues, down‐modulation of adhesion molecules and chemokine receptors, and possibly a direct impact on hematopoietic stem cells 8.

Antithymocyte globulin of horse or rabbit origin reduces the levels of antigen‐reactive T cells in the peripheral blood 4, 9. Horse ATG (hATG) is considered the standard type, and the rabbit type (rATG) is more immunosuppressive 7. Using the combination of ATG + CSA is associated with response rates of 60–80% and excellent long‐term survival among responders 4, 10, 11, 12, 13. The response to ATG and CSA typically occurs within 3 to 6 months after treatment 14.

Although both types of ATG share similar mechanisms of action, rATG may be more potent than hATG at an equivalent concentration. It can produce more profound and prolonged lymphocytopenia 15. In addition, rATG may selectively promote the expansion of regulatory T cells and induce in vivo immune tolerance through this mechanism. As a result, hATG has been the primary treatment used for severe AA, while patients with relapsing or refractory AA often receive rATG after a first course of hATG. Limited efficacy data are available for rATG used as initial therapy in severe AA. In a single‐arm phase II study, 62% of AA patients responded when treated with rATG/CSA up‐front 16. In addition, a meta‐analysis conducted in 2017 reported that a higher response rate was associated with hATG compared to rATG 17.

Manuel G. concluded that despite reports suggesting differences in biological activity of different ATG preparations, rabbit and horse ATG appear to have a similar efficacy for up‐front treatment of severe aplastic anemia 15. Similarly, Shin et al. pointed out that the efficacy of both the rATG with CSA and hATG is the same. Nevertheless, the time it took for complete response was longer with rabbit ATG 18.

According to a cohort study involving 122 patients with severe aplastic anemia, who received ATG with CSA therapy, the recorded response rates were 60%, 61%, and 58% at 3, 6, and 12 months, respectively, after initiation of treatment 9. In this particular case, the response was recorded 18 months after the initial phase of therapy.

## Conclusion

In this report, we describe a patient with severe idiopathic aplastic anemia who presented with the complaint of ecchymosis but had normal physical examination results. In examining this case, the laboratory test findings excluded the underlying potential cause. Although the patient was treated with ITS with rATG and CSA, a delayed response to treatment was witnessed 18 months later. This supports the possible delay in response to ATG treatment for patients with aplastic anemia.

## Consent

Written informed consent was obtained from the patient for publication of this case report. A copy of the written consent is available for review by the editor‐in‐chief of this journal.

## Authorship

Nujood Alzahrani, Nshwa Ashor, Traji Fathi, and Dania Bukhari participated in literature review and writing of the manuscript. Galila Zaher supervised and has made major contributions to this case report. All authors approved the final version of the manuscript.

## Conflict of Interest

None declared.
